# The Role of miR-802 in Diabetic Kidney Disease: Diagnostic and Therapeutic Insights

**DOI:** 10.3390/ijms26125474

**Published:** 2025-06-07

**Authors:** Antonio Tejera-Muñoz, Vanessa Marchant, Lucía Tejedor-Santamaría, Lucas Opazo-Ríos, Carolina Lavoz, María José Gimeno-Longas, José L. Aceña, Marta Ruiz-Ortega, Raúl R. Rodrigues-Díez

**Affiliations:** 1 Department of Endocrinology and Nutrition, General University Hospital Dr. Balmis of Alicante, Institute of Health and Biomedical Research of Alicante (ISABIAL), 03004 Alicante, Spain; 2 Health Science Faculty-HM Hospitals, Camilo José Cela University, 28036 Madrid, Spain; 3 Cellular and Molecular Biology in Renal and Vascular Pathology Laboratory, Instituto de Investigación Sanitaria-Fundación Jiménez Díaz, Universidad Autónoma de Madrid, 28040 Madrid, Spain; vanessa.marchant@uam.es (V.M.); lucia.tejedor@quironsalud.es (L.T.-S.); marta.ruiz.ortega@uam.es (M.R.-O.); 4 Facultad de Ciencias de la Salud, Universidad de Las Américas, Concepción-Talcahuano 4301099, Chile; lucasopazo78@gmail.com; 5 Unidad de Nefrología, Instituto de Medicina, Universidad Austral de Chile, Valdivia 5110566, Chile; carolavoz@gmail.com; 6 Department of Cell Biology, School of Medicine, Universidad Complutense de Madrid, 28040 Madrid, Spain; margim08@ucm.es; 7 Department of Organic and Inorganic Chemistry, Faculty of Pharmacy, University of Alcalá (IRYCIS), 28805 Madrid, Spain; jose.acena@uah.es

**Keywords:** diabetic kidney disease, miR-802, microRNAs, biomarkers, therapeutic targets

## Abstract

Diabetic kidney disease (DKD) is a serious microvascular complication of diabetes mellitus and a leading cause of end-stage kidney disease. Despite its rising incidence, awareness and early detection of renal complications remain limited. Current research in DKD aims to identify non-invasive biomarkers for early diagnosis and to develop effective therapies that go beyond controlling risk factors, as few options are available to halt or reverse kidney inflammation and fibrosis. MicroRNAs (miRNAs), key regulators of gene expression, have emerged as promising candidates for both diagnosis and treatment in DKD. Among them, miR-802 has gained attention due to its role in modulating inflammatory, fibrotic, and metabolic pathways. Elevated levels of miR-802 correlate with renal inflammation and fibrosis in diabetic and obese models, highlighting its potential as both a diagnostic biomarker and a therapeutic target. This review focuses on the emerging evidence supporting the involvement of miR-802 in the pathogenesis of DKD and its potential role as a diagnostic and therapeutic tool. In addition, considering that miR-802 has also been implicated in other diseases, such as cancer, where it may act either as a tumor suppressor or an oncogene, these contrasting effects will also be discussed as part of the broader context to better understand the multifaceted biological roles of miR-802. This review emphasizes the need for further research to clarify the molecular mechanisms of miR-802 and to assess its potential for clinical translation in DKD.

## 1. miRNAs as a Therapeutic Approach in Diabetes Mellitus and Diabetic Kidney Disease

Diabetes mellitus (DM) is a multifactorial metabolic disorder characterized by chronic hyperglycemia resulting from defects in insulin secretion, insulin action, or both [1]. The rising prevalence of type 2 diabetes (T2DM), predominantly attributable to sedentary lifestyles and obesity, has led to the classification of diabetes as a global health crisis [2]. Despite the advent of pharmacological and non-pharmacological interventions, long-term complications remain a leading cause of morbidity and mortality in diabetic patients, emphasizing the need for novel therapeutic strategies.

Although multiple factors are implicated, DM has emerged as the leading cause of chronic kidney disease (CKD) and end-stage kidney disease (ESKD) worldwide [3,4], giving rise to diabetic kidney disease (DKD). DKD is a prevalent and severe microvascular complication of DM, characterized by progressive renal damage, glomerular filtration rate decline, and culminating in ESKD. Epidemiologically, the annual incidence of ESKD due to DM is rising globally [5]. Despite the increasing prevalence of kidney disease among individuals with DM over the past two decades, awareness of kidney-related complications remains alarmingly low [6,7]. This gap in awareness, coupled with the growing burden of obesity and T2DM, creates a conducive environment for the escalation of DKD cases. Although precise data on the percentage of CKD cases caused by diabetes is lacking, it is estimated that DKD develops in nearly half of patients with T2DM and one-third of those with type 1 DM (T1DM) [3,8].

DKD primarily affects the glomerular filtration rate (GFR) and/or albumin excretion [9,10], being typically asymptomatic in its early stages and often detected through routine or periodic testing. In the early stages of DKD, especially in individuals with obesity, essential hypertension, prediabetes or borderline diabetes, and DM, glomerular hyperfiltration is an incipient mechanism that could precede renal damage [11]. This supraphysiologic increase in renal function could be measured as normal GFR (90–119 mL/min/1.73 m^2^), stage 1 hyperfiltration (120–149 mL/min/1.73 m^2^), stage 2 hyperfiltration (150–179 mL/min/1.73 m^2^) and stage 3 hyperfiltration (≥180 mL/min/1.73 m^2^) [12]. Several authors have stated that this event is very variable, occurring between 10% and 73% of individuals with T1DM and T2DM (28143897). Though the Sunder-Plassmann proposal allows GFR staging, these threshold values prevent the recognition of differences between values obtained throughout the lifespan (GFR < 149 mL/min/1.73 m^2^ is normal in young adults or age-related GFR decline), sex, ethnicity, reduced renal mass at birth, among others. New insights show that GFR can adapt to the physiological or physiopathological kidney demands. An example of this dynamic change is transient hyperfiltration following the intake of a high-protein diet, absolute hyperfiltration observed in kidneys with a normal number of nephrons, such as pregnant women, and relative hyperfiltration observed in kidneys with a reduced number of nephrons, such as solitary or single-functioning kidneys [13]. From the pathophysiological point of view, glomerular hyperfiltration causes irreversible damage to the nephron, since it increases the physiological demand of the total sum of each of the functional nephrons [14].

As a result, this event triggers the activation of signaling pathways related to proliferation, inflammation, and apoptosis, which results in injury and loss of the renal functional reserve and total number of functional nephrons [15]. Therefore, the “asymptomatic” loss of functional nephrons together with the chronicity of the work overload in residual nephrons are key drivers of the reduction in glomerular filtration rate in DKD [15]. The main sign observed in patients with DKD is podocytopathy [16]. Changes in the podocyte shape (pedicellar effacement) or in the number of podocytes (podocytopenia) alter the integrity of the glomerular filtration barrier, thickening of the basement membrane, increased mesangium, proteinuria, tubular overload, and fibrosis. For this reason, podocyte injury is a finding responsible for most of the structural changes observed at glomerular, tubular, and interstitial levels [17]. However, podocytopathy is not a pathognomonic sign of DKD and can be observed, either in isolation or in conjunction with DKD, in a variety of renal pathologies, requiring diagnostic confirmation by renal biopsy [18].

The gold standard for DKD diagnosis would precisely be kidney biopsy, although this procedure is not routinely performed in diabetic patients without proteinuria and/or advanced renal failure due to the potential risk, primarily bleeding [19]. Recent studies have reported the potential use of non-invasive biomarkers for predicting progression and evaluating the response to the treatment in DKD patients [20], although nowadays there are no validated biomarkers for clinical practice. This non-invasive diagnostic approach offers advantages in terms of convenience and safety, but the lack of biopsy data may lead to an underestimation of the true incidence and prevalence of CKD attributable to DM. Upon diagnosis, DKD is classified according to the Renal Pathology Society’s staging system, which is based on glomerular, tubulointerstitial, and vascular lesions (Table 1) [21].

Unfortunately, DKD is a non-reversible chronic pathology, so the primary goal is to slow the progression of the disease and manage its symptoms. Currently, the treatment for DKD follows the KDIGO guidelines and focuses on reducing multiple risk factors, including blood pressure control, individualized glycemic management, lipid regulation, and lifestyle modifications [22]. However, the complex pathophysiology of DKD, characterized by structural, metabolic, and functional alterations, poses significant challenges in managing and treating this condition. This issue highlights the need for new strategies that allow for early detection of the disease as well as to halt its progression as much as possible once diagnosed.

In this sense, microRNAs (miRNAs) hold significant potential in the diagnosis and treatment of DKD [23,24]. These small, non-coding RNA molecules regulate gene expression and have been implicated in the pathogenesis of DKD by modulating pathways involved in inflammation, fibrosis, and metabolic dysfunction [24]. The intricate role of miRNAs in DKD underscores their utility as both diagnostic biomarkers and therapeutic targets. In this sense, several miRNAs, including miR-21, miR-192, and miR-29, have been identified as biomarkers for the early detection of DKD, as their altered expression levels correlate with disease progression and severity [25,26,27]. The ability to detect DKD at an early stage using miRNA biomarkers could significantly improve patient outcomes by allowing for earlier intervention and more tailored treatment strategies. For example, elevated levels of miR-21 have been associated with increased renal fibrosis and inflammation, making it a potential marker for early DKD diagnosis [27,28].

In addition to their diagnostic potential, miRNAs offer promising therapeutic targets for DKD. Therapeutic inhibition of specific miRNAs can ameliorate pathological processes in the kidneys. For instance, inhibiting miR-21 has been shown to reduce renal inflammation and fibrosis in experimental models of DKD, suggesting that miR-21 inhibitors could provide clinical benefits [28,29]. The therapeutic modulation of miR-192 and miR-29 also holds potential, as these miRNAs are involved in regulating extracellular matrix production and renal fibrosis, key features of DKD pathology. It is also important to highlight the role of microRNAs in regulating glucose and lipid metabolism. Specifically, miRNAs have been shown to act as attenuators of insulin expression and secretion. In this regard, the overexpression of miR-130a, miR-130b, and miR-152 lowers the β-cell intracellular ATP/ADP ratio by targeting key enzymes, resulting in decreased insulin synthesis and secretion [30]. Among the various miRNAs studied, recent investigations—including a study published by our research group—have highlighted miR-802 as one of the most promising candidates in DKD, both as a prognostic biomarker and as a potential therapeutic target [31,32].

## 2. Current Knowledge About miR-802 in Kidney Damage: A Key Factor in DKD

MiR-802 is a miRNA encoded by a 94 bp region located in the 21q22.12 chromosomic human region, and, since its discovery, the role of this miRNA in multiple pathologies has been reported to show interesting and controversial data. Interestingly, among the centenary of canonical miRNA genes identified in the human genome, miR-802 belongs to one of the broadly conserved families from bony fish to vertebrates, being the only member of the family [33]. Specifically, in the case of humans and mice, according to the miRBase database (https://mirbase.org/, accessed on 29 November 2024), the mature sequence of miR-802 is identical in both species (hsa-miR-802 and mmu-miR-802, respectively). This high degree of sequence conservation suggests that miR-802 target genes and regulatory mechanisms observed in experimental mouse models may be extrapolated to humans, thereby supporting the translational relevance of preclinical studies involving miR-802. Although its conserved nature indicates that miR-802 may play essential roles in various biological processes, our current understanding of its biological functions remains limited.

The role of miR-802 is not yet fully understood, but research on the impact that the modulation of this miRNA may have on kidney-related disorders is currently increasing. One of the first studies relating to miR-802 and the kidney demonstrated that higher potassium intake stimulated miR-802 expression in the kidney, which targeted caveolin-1 expression, leading to increased surface expression of ROMK channels in the distal nephron [34]. Despite this postulated beneficial role of miR-802 facilitating potassium excretion, most subsequent studies suggested that the role of this miR-802 in the kidney may be deleterious. Overexpression of miR-802 in different organs has been reported to be induced by obesity in several studies. Thus, some authors demonstrated increased levels of miR-802 in the liver of obese mouse models, db/db (leptin receptor-deficient) and high-fat diet, as well as in obese human subjects [35]. At the kidney level, our group demonstrated the presence of increased levels of miR-802 in the renal cortex of obese deficient leptin mice in a diabetes susceptible strain (BTBR Ob/Ob) at 22 weeks old [31]. A common circumstance of these mouse-obese models relies on the development of diabetes and the associated nephropathy induced by high glucose levels. All these results are in line with a computational study that postulated the involvement of miR-802 in T2DM [36].

Regarding the molecular mechanisms that are modulated by miR-802 and those that modulate miR-802, there is still a long way to go. Recent studies have demonstrated that obesity increased miR-802 levels in the pancreatic islets, speculating that this increase was mediated by up-regulation of the transcription factor Foxo1 [37]. Similarly, highly increased levels of miR-802 were observed in the liver of obese and diabetic mice, but in this case, the transcription factor Hnf1b was identified as the target downregulated by miR-802 [35]. Interestingly, Hnf1b has been related to the development of maturity-onset diabetes of the young (MODY) type 5, and mutations in its sequence have also been associated with a predisposition for T2DM [38,39]. Consequently, inducible transgenic overexpression of miR-802 in mice leads to impaired insulin transcription and secretion, whereas miR-802 expression reduction improved glucose tolerance and insulin action [35,37]. The exposition of cardiac myocytes to hypertrophic adipocyte-derived exosomes, which overexpress miR-802, also resulted in insulin resistance [40], emphasizing the role of this miRNA in glucose metabolism.

A recent study has demonstrated that miR-802 can promote intercellular communication between lipid-overloaded adipocytes and macrophages [41]. In this elegant work, the authors showed that this communication triggers an inflammatory response in adipose tissue, ultimately leading to the development of insulin resistance. Through the selective overexpression of miR-802 in the adipose tissue of mice, they further demonstrated that this microRNA exacerbates adipose tissue inflammation and aggravates insulin resistance in the HFD-induced obesity model [41]. Consequently, they observed that selective depletion of miR-802 in adipose tissue improved insulin resistance and reduced inflammatory infiltrate within the tissue. Considering this strong relation between obesity, T2DM, and miR-802, several authors have studied the role of this miRNA in the development and progression of DKD, and the potential molecular mechanisms implicated. The presence of miR-802 increased levels in DM seems to be incontestable, considering studies showing increased levels of this miRNA in the serum of diabetic patients, as well as in the serum, liver, and epididymal white adipose tissue in diabetic mice [32]. Delving a bit deeper, another elegant study demonstrated that kidney-specific miR-802 silencing improves high-fat diet-induced nephropathy, improving renal dysfunction, structural disorders, and fibrosis [42]. In the same way, some studies have revealed highly increased levels of miR-802 in the kidneys of different diabetic mouse models, including the BTBR Ob/Ob and the streptozotocin-induced diabetic models [31,43]. Recently, the relevance of this miRNA as a key regulator in DKD has been corroborated by showing increased miR-802 levels in the two main compartments of the kidney, cortex, and medulla, in STZ diabetic mice [44].

Despite hyperglycemia being considered the primary driving and triggering force in the development of DKD, various evidence has also highlighted the significant involvement of the immune system and inflammation, as well as oxidative stress, in the etiology of DKD [3,45]. In this sense, miR-802 has been shown to exacerbate renal inflammation by targeting anti-inflammatory factors and promoting the expression of pro-inflammatory cytokines [42]. For instance, it has been demonstrated that miR-802 directly suppresses the NF-κB-repressing factor and positively regulates renal inflammatory response in high-fat diet-induced nephropathy [42]. Supporting these results, recent data have clearly demonstrated that miR-802 activates both the canonical and noncanonical NF-κB pathways by targeting its negative regulator, TRAF3, which in turn leads to the recruitment of macrophages [41]. Moreover, the pro-inflammatory role of miR-802 has been further substantiated by its ability to promote the polarization of M1 macrophages within adipose tissue. The mechanism regulating this polarization appears to be mediated by the indirect stimulation of SREBP1 expression via the canonical NF-κB signaling pathway [41].

Overexpression of miR-802 has also been described to increase hepatic oxidative stress and induce insulin resistance in high-fat-fed mice [46]. Some authors demonstrated that miR-802 is involved in palmitate-induced oxidative stress damage to pancreatic β cells [47]. Deleterious effects of miR-802 have also been reported in other kidney pathologies. Thus, in dogs with X-linked hereditary nephropathy, which reproduces Alport syndrome in humans and progressive CKD, miR-802 was the most upregulated one in kidneys from dogs with CKD compared to age-matched controls [48].

## 3. The Role of miR-802 in Other Pathologies: Context-Dependent Opposite Effects

One of the most studied and controversial effects of miR-802 has been reported in cancer. In these pathological situations, miR-802 exhibits a dual role, acting either as a tumor suppressor or an oncogene depending on the cellular context and the specific target genes involved. In certain types of cancer, such as gastric cancer, colorectal cancer, breast cancer, and melanoma, miR-802 is downregulated, leading to increased expression of oncogenes and promotion of tumor growth and metastasis [49]. Contrariwise, in other cancer types like hepatocellular carcinoma, bladder urothelial cancer, and osteosarcoma, miR-802 levels are increased and related to cell proliferation, migration, and invasion [49]. For instance, in osteosarcoma, miR-802 promotes the progression of fibrosis through epithelial-to-mesenchymal transition (EMT), migration, and invasion of the cells, via targeting p27 [50]. On the other hand, in gastric cancer, miR-802 acts as a tumor suppression factor by targeting RAB23 expression [51] and suppresses acinar to ductal reprogramming during the onset of pancreatic cancer by repressing oncogenic KRAS mutations [52].

Apart from DM, miR-802 has also been described to be involved in other inflammatory-related pathologies with different roles. In acute respiratory distress syndrome, miR-802 can reduce the expression of TNFα, IL1β, and IL6 in cultured monocytes under LPS stimulus, as well as in in vivo experiments, suggesting a protective role of miR-802 in this disease [53]. The essential role of miR-802 in cell function and differentiation has also been described in Paneth cells and enterocytes, respectively [33]. Thus, the absence of the miR-802 gene in the small intestine of mice induced decreased glucose uptake, impaired enterocyte differentiation, increased Paneth cell function, and intestinal epithelial proliferation. This study demonstrated miR-802 as a negative regulator of Wnt signaling, a key pathway in the activation of inflammatory processes involved in carcinogenesis and cancer cell proliferation and invasion [54], therefore suggesting a protective role of this miRNA in this case. In contrast, a recent study demonstrated that obesity-induced miR-802 promotes non-alcoholic steatohepatitis targeting AMPK in mice [55].

At the mechanistic level, miR-802 is involved in the regulation of numerous molecular processes that are critically relevant to the development of various oncogenic pathologies, including essential cellular signaling pathways such as PI3K/AKT, Wnt/β-catenin, and Hedgehog [49]. The fact that the expression of the different components of these molecular routes can be either upregulated or downregulated highlights the substantial complexity and context-dependence of therapeutic strategies that target miR-802. Moreover, it has been reported that miR-802 itself is subject to regulation by an array of non-coding RNA molecules, which introduces an additional layer of intricacy to the overall regulatory network. For instance, the circular RNA (circRNA) CircDONSON has been shown to interfere with the miR-802-mediated negative regulation of BMI1 gene expression. Under normal circumstances, miR-802 functions to inhibit BMI1, a key factor whose overexpression is known to induce resistance to chemotherapeutic agents. Indeed, elevated levels of CircDONSON have been correlated with increased cisplatin resistance in gastric cancer [56]. Similarly, the long non-coding RNA (lncRNA) IGFL2-AS1 has been observed to competitively interact with miR-802 in the regulation of ARPP19 in gastric cancer [57]. In this case, it was demonstrated that both an increase in miR-802 expression and a decrease in the synthesis of IGFL2-AS1 significantly suppressed the proliferation, migration, and invasion of gastric cancer cells in vitro, while also inhibiting tumor development in vivo. Furthermore, it has been observed that miR-802 acts as a direct target of the long non-coding RNA MIR155HG. Elevated expression levels of MIR155HG have been robustly correlated with a poor prognosis in patients diagnosed with pancreatic cancer. This association appears to be directly linked to the attenuation of miR-802’s anti-apoptotic effects in these cancer cells [58]. In addition to these regulatory interactions, miR-802 downregulates the expression of flotillin-2 (FLOT2), a protein that has been described as a promoter of tumor growth in various types of cancer [59]. In melanoma, the lncRNA MIR4435-2HG has been shown to induce cell proliferation, invasion, and migration by targeting miR-802 and thereby upregulating FLOT2 expression [58]. Thus, the context-specific behavior of miR-802 represents a significant translational challenge, and any potential therapeutic approach targeting this miRNA must be carefully tailored to avoid unintended oncogenic or suppressive effects in non-target tissues.

## 4. Key Takeaways and Future Directions

According to the KDIGO guidelines, sodium-glucose cotransporter type 2 inhibitors (SGLT2i) have been positioned as the first line of therapeutic action in people with CKD with or without DM. This positioning is based on the results of clinical studies widely disseminated worldwide (EMPA-REG, DAPA-CKD, EMPA-KIDNEY) [60,61,62]. On the other hand, the addition of GLP-1 receptor agonists or non-steroidal mineralocorticoid receptor antagonists, for now, are additional second-line therapeutic strategies in people with CKD and overweight/obesity, persistent hypertension or metabolically unstable who require much tighter clinical control for the achievement of cardio-renometabolic outcomes, with the aim of delaying renal replacement therapy [62,63].

However, in this new paradigm of therapeutic opportunities, the maintenance of residual albuminuria observed in clinical studies suggests that there is still an important therapeutic margin. In this context, GLP-1 receptor agonists and SGLT2i have been correlated with RNA-like molecules, particularly microRNAs (miRNAs). Several authors have proposed miRNAs as a strategy to enhance the ability to predict the effectiveness of antidiabetic drugs [64], as they can regulate numerous genes associated with type 2 diabetes (T2DM). Various miRNAs have been suggested to regulate GLP-1 receptor signaling [65,66] and the action of SGLT2i [67]. Therefore, studies on miRNAs may converge into promising strategies for predicting responses to diabetes medications, enabling the design of specific pathways and advancing step-by-step toward more personalized medicine.

**Figure 1:**
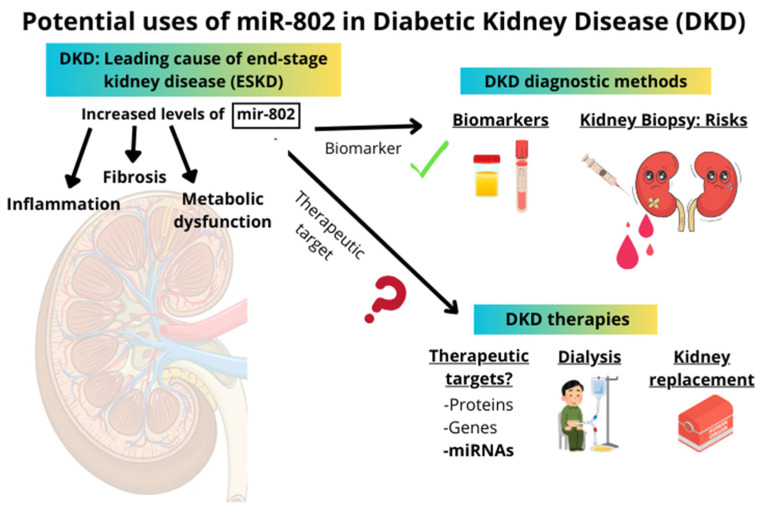
The right side of the image presents a schematic overview of current diagnostic methods and therapeutic approaches in diabetic kidney disease (DKD). The left side illustrates the potential applications of miR-802 as a biomarker or therapeutic target in DKD. miR-802 is suggested as a promising candidate biomarker; however, its role as a therapeutic target remains uncertain and requires further investigation.

Currently, there are some clinical trials (phases I and II) incorporating the use of miRNAs [68]. However, the translation of miRNAs into clinical practice as therapeutic targets remains quite distant, primarily due to the multiple targets of certain miRNAs and their varied effects depending on the context in which they are expressed. Conversely, the use of miRNAs as diagnostic and prognostic biomarkers is currently employed in several pathological situations [68,69]. Considering all the above-mentioned studies about miR-802 in different pathologies, miR-802 could be an excellent potential biomarker for kidney damage in several conditions, primarily in DKD. However, miR-802 plays a critical role in both inflammation and cancer processes, exerting context-dependent effects on gene expression and cellular functions. This highlights the need for more detailed studies before using miR-802 as a potential therapeutic target in DKD (Figure 1).

**Conceptual Keys**

- DKD is a major complication of diabetes mellitus and the leading cause of end-stage kidney disease (ESKD).
- The primary research objective is to find biomarkers for early diagnosis and potential therapies for DKD, with miRNAs being potential options for both.
- Elevated levels of miR-802 in both serum and tissue-specific levels of DM complications (kidney, liver, and adipose tissue) correlate with inflammation and fibrosis in mouse models.
- Growing evidence suggests that it could be a potential early, non-invasive biomarker for DKD.
- However, miR-802 acts in a dual role as a protective or deleterious factor in different diseases, mainly in cancer.
- miR-802 could be a promising biomarker for early DKD diagnosis, but more research is required to fully understand miR-802’s therapeutic potential in DKD.

## Figures and Tables

**Table 1:** Histopathological classification of morphological changes in diabetic kidney disease.

Glomerular Lesions		Tubulointerstitial Lesions		Vascular Lesions	
Class	Description	Interstitial Fibrosis and Tubular Atrophy (IFTA)		Arteriolar Hyalinosis	
I	Glomerular Basement Membrane Thickening	**Score**	**Description**	**Score**	**Description**
IIa	Mild Mesangial Expansion	0	Absent	0	Absent
IIb	Severe Mesangial Expansion	1	<25%	1	At least one arteriole
III	Nodular Sclerosis with presence of Kimmelstiel–Wilson lesions	2	25–50%	2	More than one arteriole
IV	Advanced Diabetic Glomerulosclerosis	3	>50%		
		**Interstitial Inflammation**		**Arteriosclerosis (Worst Artery Assessment)**	
		0	Absent	0	Without intimal thickening
		1	Infiltration with IFTA	1	Intimal thickening < Media thickening
		2	Infiltration without IFTA	2	Intimal thickening > Media thickening

## Abbreviations

The following abbreviations are used in this manuscript:

DKD	Diabetic Kidney Disease
ESKD	End-Stage Kidney Disease
miRNAs	MicroRNAs
CKD	Chronic Kidney Disease
DM	Diabetes Mellitus
T2DM	Type 2 Diabetes Mellitus
T1DM	Type 1 Diabetes Mellitus
STZ	Streptozotocin
NF-κB	Nuclear factor kappa B
TNFα	Tumor necrosis factor alpha
IL1β	Interleukin 1 beta
IL6	Interleukin 6
LPS	Lipopolysaccharide
EMT	Epithelial-to-Mesenchymal Transition
AMPK	5′ AMP-activated protein kinase
GLP-1	Glucagon-like peptide-1
